# Annotations

**Published:** 1902-07-26

**Authors:** 


### Annotations.

### Red Tape in Army Medical Affairs.

When Mr. Richards, on the vote for the medical establishment, drew the attention of the Secretary for War to the complicated formulæ, the signing and the countersigning of orders, which have to be gone through before medical appliances, necessary for the treatment of the sick and wounded in war, could be obtained from the stores, and when he enforced his demand for reform in the system of accounts by referring to what happened at Bloemfontein, he obviously laid himself open to the retort that he was dealing with matters of ancient history, for, as Mr. Brodrick said, "immense progress and amelioration had taken place" in the last two years. Nevertheless, the question put was an important one, and the assurance asked for was curiously slow in coming. At the time of the Bloemfontein scandals much was made of the lack of transport and of the difficulty of bringing up hospital stores from the base. All this, however, was largely beside the point. The real scandal was that the stores were actually there; that notwithstanding all the difficulties the blankets had been brought up, and yet that the soldiers were lying on the bare ground because of some red-tape arrangement or lack of arrangement which stood in the way of the distribution of the blankets among the hospitals where they were required. Mr. Richards did well, then, in sticking to his point and repeating his question: "Whether he intended to continue the system of requiring a number of signatures for medical stores, practically on the battlefield, when men were dying for want of articles which were within 20 yards of them?" In the end, after Colonel Welby had moved a reduction of the vote by £100, Mr. Brodrick stated that he was giving his attention to the simplification of all medical forms, and that a departmental committee, under Sir William Butler, had been appointed to that end. Why this simple answer could not have been given at first we are at a loss to see. The things we want for ourselves, speaking professionally, are pay, position, and opportunities for gaining knowledge and experience. But what we want for the service is that it shall be efficient in times of stress and danger; and this it can never be so long as such a system of signing and countersigning of orders for medical necessaries, as was described by Mr. Richards, remains in operation.

### Tuberculosis in Asylums.

The report of the Tuberculosis Committee appointed by the Medico-Psychological Society in regard to the prevalence of phthisis in asylums forms a very serious indictment of the management of these institutions. It has long been known that the death-rate from phthisis among asylum patients was far higher than among persons of similar ages outside, but when we find it authoritatively stated that not only is this the case but that a large proportion of the patients so affected contract their disease after their admission into asylums—institutions specially built and designed for their safe keeping—we need not hesitate to insist on the gravity of the situation. What is the root of the mischief is pretty clearly indicated in the report. The committee is satisfied that deficiency of air space, or in other words overcrowding, is the most important factor in the production of this unfortunate state of affairs. We are of opinion, say the writers of the report, "that the predisposition to, and the actual infection of, phthisis is to a very large extent started in the sleeping rooms of our overcrowded asylums," and they go on to recommend that an air space of 1,200 cubic feet with efficient ventilation should be the minimum provided for each sleeping patient. Needless to say that to carry out any such recommendation means at once a very heavy increase in the cost of providing asylum accommodation for the ever-growing numbers of the insane. We cannot but feel however that the management of asylums as well as their construction has a good deal to do with the matter. Knowing what we now know about undesirability of keeping living rooms permanently warm, about the advantages of sleeping with widely open windows, about the health giving properties of fresh air, and about the deteriorating influence of indoor life, it is impossible not to suspect that the tenderness of these lunatics is largely due to their being kept as the gardeners would say "under glass," and that what is wanted is not merely more air space but more constantly open windows, and many more hours a day spent in the open. Acute insanity is a thing more or less apart, like acute disease; but chronic lunatics should be well fed and well clad, and above all things they should be got out of doors and kept occupied in the fresh air.

### Soldiers and their Feet.

The recent war seems to have taught the War Office the importance of the soldiers' feet. So much we infer from the announcement that an order has been issued providing that a soldier, not above the rank of a sergeant, may be employed as a chiropodist in each infantry battalion, provided he has undergone a course of instruction for one month, and obtained a certificate of proficiency in chiropody. The chiropodist is not to be excused any of his ordinary duties, but as a reward for his extra work is to receive extra pay at the rate of sixpence a day for six days of the week. Whether or not a soldier with a month's training, who fills up the leisure left by his ordinary work by cutting his comrades' corns, is all the Army needs in the way of a chiropodist may not be certain, but at least the order shows the consciousness that much of the success of a campaign may depend on the marching capacity of the soldiers, and that no man can march well and long whose every step is made painful by corns or some other ailment of the foot. It is well to remember, however, that prevention is better than cure, and that the battalion cobbler and the contractor who provides the Army boots are persons to be carefully selected and as carefully watched. In the German Army, where the feet of the soldiers are appreciated at their true importance, foot-rags—a sort of bandage which can be wound round each of the toes and round the feet—are often more popular than socks. These rags absorb the perspiration and prevent the toes rubbing against each other. Hence they are a boon on long marches. Moreover they are cheap, and a supply of them can be carried more easily than a sufficiency of socks.

